# The Relationship between Fixation Stability and Retinal Structural Parameters in Children with Anisometropic, Strabismic and Mixed Amblyopia

**DOI:** 10.3390/life13071517

**Published:** 2023-07-06

**Authors:** Raquel Mompart-Martínez, Marc Argilés, Genis Cardona, Lluís Cavero-Roig, Lluís González-Sanchís, Maria Soledad Pighin

**Affiliations:** 1 Institut Català de la Retina (ICR), 08022 Barcelona, Spain; mompart.r@gmail.com (R.M.-M.); lluis.cavero@icrcat.com (L.C.-R.); lgonzalez@icrcat.com (L.G.-S.); solepin@gmail.com (M.S.P.); 2 Department of Optics and Optometry, Universitat Politècnica de Catalunya (UPC), 08222 Terrassa, Spain; marc.argiles@upc.edu; 3 Center for Sensors, Instruments, and Systems Development (CD6), Universitat Politècnica de Catalunya (UPC), 08222 Terrassa, Spain; 4 Applied Optics and Image Processing Group (GOAPI), Universitat Politècnica de Catalunya (UPC), 08222 Terrassa, Spain

**Keywords:** amblyopia, fixation stability, retinal microvasculature, macular thickness, macular volume, stereoacuity, strabismus, anisometropia

## Abstract

(1) Background: Amblyopia is an ocular condition leading to structural and functional changes. The relationship between these changes is complex and remains poorly understood. (2) Methods: Participants included 31 children aged 5 to 9 years with strabismic (*n* = 9), anisometropic (*n* = 16) and mixed (*n* = 6) unilateral amblyopia, and 14 age-matched non-amblyopic children. The 95% and 63% Bivariate Contour Ellipse Area (BCEA), axial length, Foveal Avascular Zone (FAZ) area, center macular thickness and volume were assessed. The relationship between these parameters was explored. (3) Results: Statistically significant differences were found among the four groups in best corrected distance visual acuity (BCVA) (*p* < 0.001), BCEA 95% (*p* = 0.002) and BCEA 63% (*p* = 0.002), but not in the FAZ area, central macular thickness, central macular volume and axial length. Eyes with amblyopia had poorer BCVA and larger fixation instability than controls. Inter-ocular differences were more significant in patients with strabismic amblyopia, particularly in BCVA (*p* = 0.003), central macular thickness (*p* < 0.001) and central macular volume (*p* = 0.002). In amblyopic eyes, BCEA 95% and 63% were correlated with BCVA, but not with the FAZ area. (4) Conclusion: Amblyopia is associated with a reduction in fixation stability and BCVA, although there is a general lack of correlation with structural changes, suggesting a complex interaction between anatomy and function in amblyopia.

## 1. Introduction

Amblyopia is a neurodevelopmental disorder that results from an abnormal visual experience during a critical period of visual development [1,2]. Risk factors that typically contribute to and serve to classify amblyopia include strabismus (strabismic amblyopia), anisometropic uncorrected refractive error (anisometric amblyopia) or a combination of both (mixed amblyopia) [2,3]. Several studies have reported possible structural changes in the visual cortex [4] and lateral geniculate nucleus [5] in amblyopic eyes, compared with normal eyes [6], as well as differences in macular thickness and volume [7,8], retinal nerve fiber layer (RNFL) [8,9], and macular capillary vascular structure, including the foveal avascular zone (FAZ) area [10,11,12,13,14].

Current developments in microperimetry have allowed researchers to explore fixation stability in amblyopia [1,15,16,17,18,19,20,21] and the relationship between fixation stability, visual acuity and stereoacuity in different types of amblyopia, which remains poorly understood [15,16]. The analysis of fixation stability has also been employed to assess the effectiveness of different types of treatments for amblyopia, such as part-time occlusion of the non-amblyopic eye [22,23,24] or surgical intervention to correct strabismus [25].

Previous researchers have explored the association between retinal structural parameters, such as the FAZ area, and visual function parameters, including visual acuity, visual fields and fixation stability, in retinal vein occlusion, diabetic retinopathy and other pathologies [26,27,28]. However, the relationship between fixation stability and specific retinal anatomical features in various types of amblyopia is not conclusive. Similarly, several studies have investigated differences in the FAZ area between amblyopic and contralateral eyes, as well as healthy controls, reporting contradictory evidence [10,12,29,30,31,32]. These discrepancies may be due to variations in sample demographics, such as age (adults or children) and distribution of amblyopia types, as well as differences in instrumentation, analysis software and methodology. Although this previous research has shown vascular anomalies in amblyopic patients in terms of density and structure [10,12], less attention has been paid to the possible relationship between fixation stability, as determined by the bivariate contour ellipse area (BCEA) and the FAZ area. Indeed, although one study failed to find any correlation between these parameters in patients with retinopathy and prematurity [28], to the best of our knowledge, it has not been studied in amblyopia and different subtypes of strabismus. As previous researchers have observed different patterns of visual function depending on the degree and type of amblyopia [33], it may be relevant to explore fixation stability and structural parameters in different subtypes of amblyopia and strabismus.

Thus, it was the aim of the present study to assess the possible correlation between fixation stability, defined by BCEA, and structural parameters, such as the FAZ area, in a sample of children with unilateral strabismic, anisometropic and mixed amblyopia. Additional study variables were axial length (AL), best corrected distance visual acuity (BCVA) and stereoacuity, as well as central macular thickness and volume. Results from the amblyopic eyes were compared with those of the contralateral eye, and with those of a non-amblyopic control group.

## 2. Materials and Methods

### 2.1. Study Sample

A cross-sectional study was conducted at the Institut Català de la Retina (ICR, Barcelona, Spain) from September 2021 to May 2022. The study was approved by the Ethics Committee of the Grupo Hospitalario Quirón Salud, (ICR-13/21-2022/10-OFT-ICR) and was compliant with the principles of the Declaration of Helsinki. Informed consent was obtained from legal guardians after a detailed explanation of the nature and possible consequences of the study.

Children aged 5 to 9 years were recruited for this investigation. Participants in the study had either strabismic, anisometropic or mixed unilateral amblyopia. Amblyopia was defined as BCVA of ≥0.2 logMAR for the worse eye and an interocular difference of ≥0.15 logMAR. In addition, for anisometropic amblyopia, interocular differences in refractive error needed to be of 2.00 diopters (D) or more in sphere and/or 1.50 D in cylinder. No anisohyperopic participants were included in the study. In addition, strabismic amblyopia required a minimum angle of manifest deviation of 5 prism diopters. Patients diagnosed with neurological pathologies, retinopathies and/or maculopathies, glaucoma, nystagmus, media opacities, systemic diseases, cardiovascular or renal diseases, retardation and/or prematurity were excluded from the study, as were those with a manifest deviation of 30 prism diopters or more. In addition, a control age and gender-matched group of healthy non-amblyopic subjects was included.

### 2.2. Procedure

Monocular BCVA was evaluated with the Early Treatment Diabetic Retinopathy Study (ETDRS) test at 4 m and recorded letter by letter in logMAR notation. Stereoacuity was evaluated with the TNO stereo test, with the aid of red–green filters (a score of 800 arc seconds was assigned to patients failing to identify any of the pictures). For both BCVA and stereoacuity, patients received instructions on the procedure and an initial trial was conducted to ensure they fully understood what was expected of them. For this trial, the largest letter of the ETDRS test was used for BCVA measurements, and the butterfly plate of the TNO test for stereoacuity, which corresponds to 1500 arc seconds. Exactly the same procedure was employed for all patients and all measurements were conducted by an examiner unaware of the patient group allocation, although in patients with strabismic amblyopia this single-blind experimental condition could not be maintained. All patients included in the study were able to follow the BCVA and stereoacuity measurement procedures without difficulties.

Refractive error was measured using the retinoscopy technique, with and without cycloplegia. Visual alignment was measured with the cover test in both near and far vision, and the angle of deviation of strabismic patients was determined with the cover test and a prismatic bar, both in conditions of best corrected visual acuity.

**Figure 1:**
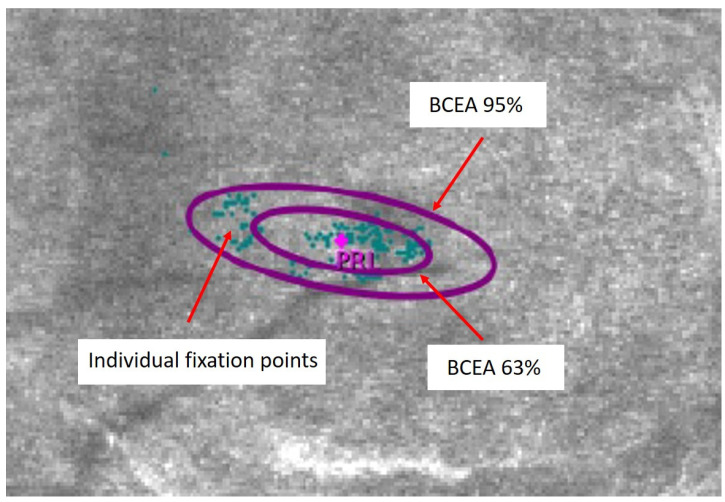
Best fit elliptic contour containing either 95% (BCEA 95%) or 63% (BCEA 63%) of the fixation points in a patient with strabismic amblyopia. PRL: Preferred Retinal Locus.

Fixation stability was measured using the Macular Analyzer Integrity Assessment (MAIA) microperimeter (CenterVue, Padova, Italy) and followed the BCEA methodology described by Crossland and co-workers, with a fixation strategy of 30 s [34]. Briefly, for each eye, assessment of fixation stability begins with the capture of a reference fundus image and the identification by the examiner of a high contrast macular retinal landmark. During the subsequent 30 s test period, the patient is instructed to look at the fixation stimulus (red dot) while the MAIA software (version 2.6.0) determines the shift between the reference image and the real-time fundus image at 25 Hz, obtaining 750 sets of coordinates (X and Y) to describe fixation changes. The software then defines the BCEA as the best fit elliptic contour containing either 95% (BCEA 95%) or 63% (BCEA 63%) of the fixation points, with smaller BCEA values denoting better fixation stability (see, for example, Figure 1). The MAIA automatically corrects for refraction errors in the spherical equivalent range of −15.00 to +10.00 D, allowing for the measurements to be conducted without habitual refractive correction.

**Figure 2:**
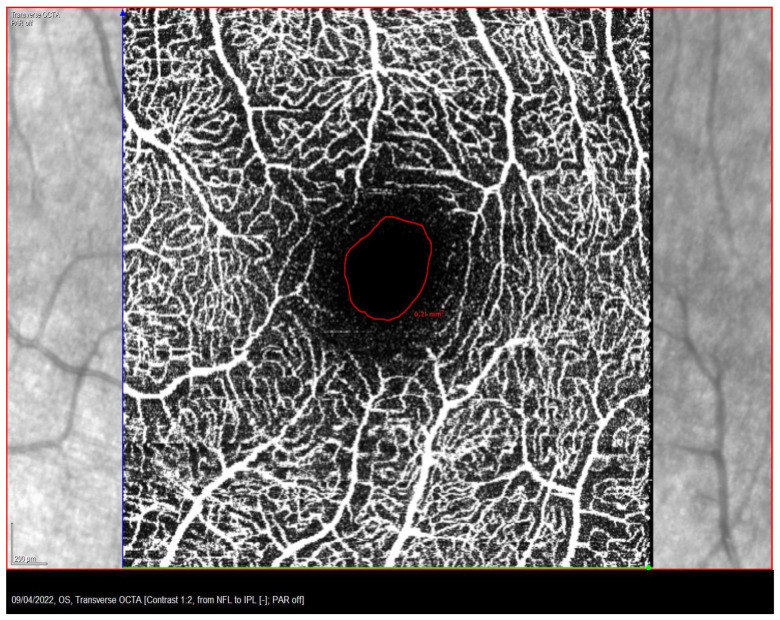
Manually delimited foveal vascular zone (FAZ) area in a patient with strabismic amblyopia.

The FAZ area of the superficial capillary plexus (SCP) was measured with the OCT-A Spectralis (Heidelberg Engineering, Heidelberg, Germany) with angiography software. To determine the FAZ area, a scan pattern of 10 × 10 degrees (consisting of 512 scanned sections separated by 6 µm) was centered on the fovea. Internal fixation was used to ensure proper alignment of the eye. It may be noted that in certain patients with large fixation instability, internal fixation may be eccentric instead of central. However, if the fixation moved outside of the initial internal fixation zone, the OCT stopped the test until the patient correctly fixed the stimulus again. Each scan was automatically segmented. Finally, the FAZ area was calculated manually employing the tools provided by the Spectralis software: the boundaries of the FAZ area were delineated by the freehand tool and calculated in mm^2^, as previously described (Figure 2) [35]. Axial length (AL) was measured with the ZEISS IOLMaster 700 biometer (Carl Zeiss Meditec, Jena, Germany).

Macular thickness and volume were measured using the OCT-A Spectralis. A macular cube scan pattern of 20 × 15 degrees (consisting of 512 HR A-scans, 37 sections separated by 120 μm) centered on the fovea was acquired. Internal fixation was used for proper alignment of the eye. Each scan was automatically segmented and an optometrist examined all macular cubes to ensure that the analyzed macular thickness and volume corresponded to the foveal center.

### 2.3. Data Analysis

SPSS version 27 (IBM Corp., Armonk, NY, USA) for Windows was used for data analysis. Values of BCEA were transformed to their corresponding log_10_ values. The Shapiro–Wilk test was employed to explore data normality, whereupon results are accordingly described as mean and standard deviation (SD), or median and range. Inferential analysis was conducted with the ANOVA or Kruskal–Wallis tests, with the corresponding post-hoc Bonferroni or Dunn–Bonferroni pair-wise analysis and correction to account for multiple comparisons, or with the paired Student’s *t*-test or Wilcoxon tests. In addition, possible associations between variables were analyzed with the Spearman coefficient of correlation test, and the Chi-Squared test was employed for frequency distribution analysis. A *p*-value less than 0.05 was considered to denote statistical significance.

Sample size calculation was performed using G*Power version 3.1.9.2 software (Heinrich-Heine-University Dusseldorf, Dusseldorf, Germany). The study of Subramanian and co-workers [15] was used as a reference for sample size calculation. The main outcome for this calculation was BCEA, with an estimated common SD of 0.38 log deg^2^ and a minimum expected difference between amblyopic and fellow eyes of 0.36 log deg^2^, and considering a 95% statistical power. The required minimum sample size was 18 participants per group for pair-wise comparisons.

## 3. Results

A total of 31 children were included in the amblyopic group (15 boys, 14 girls) aged 5 to 9 years (mean ± SD of 6.6 ± 1.1 years), with strabismic (*n* = 9), anisometropic (*n* = 16) and mixed (*n* = 6) amblyopia. The control group included 14 non-amblyopic children (7 boys, 7 girls), aged 6 to 9 years (7.6 ± 1.0 years). No statistically significant difference was found in neither age nor sex distribution between the three amblyopic and control groups (*p* > 0.05).

For the analysis, first eyes with different types of amblyopia were compared amongst them and with one of the eyes of subjects from the control group. Table 1 presents a summary of the results of monocular BCVA, stereoacuity, log BCEA 95%, log BCEA 63%, FAZ area, central macular thickness, central macular volume and axial length of strabismic, anisometropic and mixed amblyopic eyes. To compare with amblyopic eyes, the non-dominant eye was selected from the control group, except for stereoacuity measurements. Statistically significant differences were found among the four groups in BCVA (*p* < 0.001), stereoacuity (*p* < 0.001), BCEA 95% (*p* = 0.002) and BCEA 63% (*p* = 0.002), but not in the FAZ area, central macular thickness, central macular volume and axial length (all *p* > 0.05). A pair-wise analysis with the post-hoc Dunn–Bonferroni test revealed statistically significant differences in BCVA and stereoacuity between control and strabismic, anisometric and mixed amblyopic groups (all *p* < 0.001), with superior values of both visual function parameters in the control group. Regarding BCEA 95% and BCEA 63%, the Dunn–Bonferroni or Bonferroni tests revealed statistically significant differences between control eyes and strabismic and anisometropic eyes (*p* = 0.001 and *p* = 0.034, respectively, for BCEA 95%; *p* = 0.004 and *p* = 0.018, respectively, for BCEA 63%), but not between mixed amblyopic and control eyes. Strabismic eyes tended to have the largest BCEA 95% and BCEA 63% results. By pooling the data from all amblyopic groups, statistically significant differences were found between BCEA 95% and BCEA 63% (*p* < 0.001).

For the second part of the analysis, inter-ocular differences between the amblyopic eye (or non-dominant eye in controls) and the dominant eyes were calculated for amblyopic patients and control subjects. Table 2 presents a summary of the inter-ocular differences in functional and structural parameters. Statistically significant inter-ocular differences were predominant in patients with anisometropic amblyopia (BCVA, *p* = 0.003; central macular thickness, *p* < 0.001; central macular volume, *p* = 0.002). No statistically significant interocular difference was found in control subjects.

Inter-ocular differences amongst groups in BCVA, BCEA 95%, BCEA 63% and central macular thickness were statistically significant (*p* = 0.007, *p* = 0.043, *p* = 0.026 and *p* < 0.001, respectively). Post-hoc pair-wise analysis revealed statistically significant inter-ocular differences in BCVA between the anisometropic and control groups (*p* = 0.007) and between the mixed and control groups (*p* = 0.018); in BCEA 95% between anisometropic and control groups (*p* = 0.033); in BCEA 63% between the strabismic and control groups (*p* = 0.042), and between the anisometropic and control groups (*p* = 0.045); and in central macular thickness between all three amblyopic groups and the control group (strabismic, *p* = 0.013; anisometropic, *p* < 0.001; mixed, *p* = 0.020).

By pooling the data from all amblyopic groups, and considering only the amblyopic eyes, statistically significant moderate correlations were found between BCVA and both BCEA 95% (ρ = 0.575, *p* < 0.001) and BCEA 63% (ρ = 0.580, *p* < 0.001). BCEA 95% and 63% showed a very strong correlation (ρ = 0.987, *p* < 0.001). Statistically significant moderate to strong correlations were found in the non-dominant eyes of the control group between the FAZ area and both central macular thickness (ρ = −0.562, *p* < 0.001) and central macular volume (ρ = −0.740, *p* < 0.001). However, in the amblyopic groups, only a weak correlation was evidenced between the FAZ area and central macular thickness (ρ = −0.378, *p* = 0.012). Upon examining each amblyopic group independently, the only significant correlation was between BCVA and central macular thickness in the strabismic group (ρ = −0.857, *p* = 0.024), as well as between both BCEA outcomes. No statistically significant correlation was evidenced in any group between BCEA 95% or BCEA 63% and the FAZ area. In particular, in strabismic and mixed amblyopia, there was no correlation between angle of deviation and fixation stability. In addition, inter-ocular differences in BVCA in amblyopic eyes were weakly correlated with inter-ocular differences in BCEA 95% (ρ = 0.313, *p* = 0.039) and BCEA 63% (ρ = 0.321, *p* = 0.036) and moderately correlated with inter-ocular differences in central macular thickness (ρ = 0.447, *p* = 0.003).

## 4. Discussion

The aim of the present study was to explore BCVA, stereoacuity, fixation stability and retinal structural parameters (FAZ area, central macular thickness and volume), as well as AL, in children with different types of amblyopia and in normal controls. The relationship between structural and functional parameters was investigated.

Fixation stability, determined with the BCEA 95% and 63% was found to be particularly compromised in strabismic and, to a less extend, in anisometropic amblyopia, as compared with control eyes. No difference between control eyes and those with mixed amblyopia was found, although the reduced sample size for this type of amblyopia led to a slightly underpowered analysis, thus increasing the probability of type I error. These findings are in agreement with previous reports [15,19]. For instance, with the research of Subramanian et al. (2013), who also documented a reduction in stereoacuity related to amblyopia [15], as described in the present study. These authors attributed the discrepancies in the BCEA values between their study and published literature to the actual BCEA percentage under analysis [15,19]. To our knowledge, no previous research has explored both BCEA 95% and BCEA 63% from the same sample of patients. Although BCEA 95% and BCEA 63% were found to present a very strong correlation, a statistically significant difference was found between their values, underlining the need to interpret data with caution when comparing studies using different BCEA percentages. In addition, discrepancies in BCEA values among studies may be accounted for by the differences in age of participants and instrumentation.

To determine fixation stability, a fixation strategy of 30 s was implemented, which is the current minimum available interval for the MAIA microperimeter to provide reliable measurements. Given the age of some of the participants, they needed encouragement to maintain fixation on the target stimulus (for instance, they were instructed to carefully check the red fixation stimulus and tell the examiner immediately if it changed color to green). Previous researchers have employed longer fixation strategies, of 45 s or even 1 min intervals [36]. It must be highlighted that the software of MAIA constantly compares the fixation of the patient with the initial reference point defined by the examiner and automatically stops the measurement if fixation is lost or if the patient performs intrusive saccades, renewing measurements once the patient has gained the initial fixation reference. Although differences in instrumentation and sample characteristics may advise against any direct comparison, the variance in BCEA values of the present sample of young children is comparable to that obtained in previous research in adults, which may reflect that measurements in children are no less reliable than in adults, given the necessary precautions are taken [37,38].

No statistically significant differences were found in the FAZ area between amblyopic and control eyes, albeit a trend was observed in which anisometropic and mixed amblyopic eyes had larger FAZ areas than strabismic and control eyes. These findings would be in agreement with previous literature documenting larger FAZ areas in the SCP layer in anisometropic amblyopia [29,30], albeit other researchers have noted smaller FAZ areas in patients with anisometropic amblyopia, with or without strabismus [12], and in strabismic amblyopia [32].

Upon examining inter-ocular differences in functional and structural parameters, that is, amblyopic eye versus the contralateral eye, or both eyes of the control group, statistically significant differences were predominant in patients with anisometropic amblyopia, particularly in BCVA, central macular thickness and central macular volume. Inter-ocular differences in BCEA 95% and BCEA 63% were larger in strabismic and anisometropic patients than in the control group. Similarly, inter-ocular differences in central macular thickness were more noticeable in the three amblyopic groups than in the control group. These findings suggest that the study of inter-ocular asymmetry in functional and anatomical parameters may be valuable.

Correlation analysis revealed several statistically significant, moderate correlations among some functional parameters, as well as among several anatomical parameters, but no correlation was disclosed between functional and anatomical parameters, with the exception of BCVA and central macular thickness in the strabismic group. Thus, for instance, whereas in amblyopia both BCEA values were moderately correlated with BCVA, BCEA and the FAZ area did not display any significant correlation, which may suggest that the FAZ area does not influence fixation stability, although further research is needed to confirm this hypothesis. Indeed, the lack of correlation between fixation stability and the FAZ area has been previously reported for other retinal pathologies [26,27,28], but, as far as we know, it has not been explored in amblyopia. Similarly, the FAZ area was not correlated with BCVA, in contrast with the report by Huang et al. (2021) [31], in which the authors observed an improvement in both visual acuity and FAZ area following 6 months of amblyopia treatment.

This study had some limitations, mainly a reduced sample size and slightly underpowered analysis, considering a required estimated sample size of 18 per group. Unfortunately, these patients are relatively young and sometimes found it difficult to cooperate with the measurements and could not be included in the study. In cases of wide-angle strabismus, certain tests could not be performed due to saccadic re-fixational eye movements that prevented correct captures for scans. In addition, the manual nature of some of these measurements may contribute to reducing the reproducibility of some of the findings. Thus, FAZ area measurements were performed manually, following the border of the vascular and the avascular textures shown in the OCT-A image, as previously described [35]. There are some OCT-A devices with integrated software for quantitative analysis, such as the OCT 5000 (Zeiss Meditec Inc., Dublin, CA, USA) or the RTVue XR Avanti (Optovue Inc., Fremont, CA, USA). When this possibility is not available, third-party software, such as the Fiji toolbox, could be used to assist in this analysis.

In addition, although the TNO test has been documented to be adequate to measure stereoacuity in children aged 3 to 6 years old [39], there are currently other objective methods to estimate stereoacuity which may be implemented in future studies. For instance, a promising avenue of research, which has recently proved its clinical applicability in adults and children, is the analysis of ocular-following responses through video-oculography [40,41]. Briefly, ocular-following responses exhibit a strong binocular summation, which is sensitive to the interocular correlation between stimuli presented under binocular conditions, mediated by disparity-sensitive cortical neurons. It is therefore assumed that in patients with impaired stereoacuity, such as in amblyopia, ocular-following responses are compromised.

In conclusion, children with amblyopia have more fixation instability compared to normal controls. Although BCEA 95% and 63% show a strong correlation, these values are not interchangeable. Fixation instability is associated with reduced visual acuity, but not with the FAZ area, suggesting a complex interaction between structural and functional changes in amblyopia, which supports further investigation.

## Figures and Tables

**Table 1:** Best corrected distance visual acuity (BCVA), stereoacuity, log BCEA 95%, log BCEA 63%, FAZ area, central macular thickness, central macular volume and axial length for strabismic, anisometropic and mixed amblyopic eyes and control eyes (one eye per control subject was selected, except for stereoacuity evaluation). Results are shown as either mean ± SD or median (range) according to the normality of their distribution. The outcome of the ANOVA or Kruskal–Wallis tests (according to data normality) is displayed in the rightmost column.

	Strabismic Amblyopia	Anisometropic Amblyopia	Mixed Amblyopia	Control	*p*-Value
BCVA (logMAR)	0.362 ± 0.267	0.250 (0.800)	0.450 ± 0.356	0.000 (0.100)	<0.001
Stereoacuity (arc seconds)	400 (700)	200 (720)	800 (400)	40 (15)	<0.001
Log BCEA 95% (log deg^2^)	0.323 ± 0.496	0.079 ± 0.354	0.110 ± 0.425	−0.316 ± 0.255	0.002
Log BCEA 63% (log deg^2^)	−0.143 ± −0.496	−0.391 ± 0.336	−0.397 ± 0.467	−0.699 (0.602)	0.006
FAZ area (mm^2^)	0.270 ± 0.173	0.369 ± 0.190	0.354 ± 0.113	0.269 ± 0.112	0.277
Central macular thickness (µm)	269.0 (83.0)	269.4 ± 20.0	251.5 ± 23.7	261.1 ± 27.6	0.496
Central macular volume (µm^3^)	0.21 ± 0.01	0.22 ± 0.01	0.21 ± 0.01	0.22 (0.37)	0.291
Axial length (mm)	21.86 ± 0.62	22.34 ± 1.79	22.22 (6.41)	22.47 ± 1.20	0.563

**Table 2:** Inter-ocular differences in best corrected distance visual acuity (BCVA), log BCEA 95%, log BCEA 63%, FAZ area, central macular thickness, central macular volume and axial length for amblyopic and control groups (determined as amblyopic eye minus dominant eye in the amblyopic groups, or non-dominant eye minus dominant eye in the control group). Results are shown as either mean ± SD or median (range) according to the normality of their distribution, together with the outcome of the paired Student’s *t*-test or the Wilcoxon test (according to data normality).

	Strabismic Amblyopia	Anisometropic Amblyopia	Mixed Amblyopia	Control
BCVA (logMAR)	0.189 ± 0.226	0.194 ± 0.191	0.333 ± 0.367	−0.007 (0.100)
	*p* = 0.050	*p* = 0.003	*p* = 0.106	*p* = 1.000
Log BCEA 95% (log deg^2^)	0.235 ± 0.380	0.240 ± 0.433	0.003 ± 0.449	−0.052 (0.862)
	*p* = 0.097	*p* = 0.066	*p* = 0.855	*p* = 0.132
Log BCEA 63% (log deg^2^)	0.288 ± 0.421	0.236 ± 0.424	−0.069 ± 0.428	−0.038 (0.778)
	*p* = 0.058	*p* = 0.086	*p* = 1.000	*p* = 0.418
FAZ area (mm^2^)	−0.025 (1.060)	0.033 ± 0.091	−0.002 ± 0.081	−0.007 (0.130)
	*p* = 0.310	*p* = 0.105	*p* = 1.000	*p* = 0.571
Central macular thickness (µm)	16.7 ± 17.2	19.1 ± 14.8	21.0 (19.0)	−0.143 (22.0)
	*p* = 0.025	*p* < 0.001	*p* = 0.063	*p* = 1.000
Central macular volume (µm^3^)	0.01 ± 0.01	0.01 (0.03)	0.01 ± 0.01	0.00 (0.37)
	*p* = 0.073	*p* = 0.002	*p* = 0.181	*p* = 0.098
Axial length (mm)	−0.12 ± 0.19	−0.26 (6.5)	0.64 ± 1.5	0.00 (0.33)
	*p* = 0.074	*p* = 0.438	*p* = 1.000	*p* = 0.483

## Data Availability

Data may be available upon reasonable request to the authors.
